# Role of Effector-Sensitivity Gene Interactions and Durability of Quantitative Resistance to Septoria Nodorum Blotch in Eastern U.S. Wheat

**DOI:** 10.3389/fpls.2020.00155

**Published:** 2020-03-06

**Authors:** Christina Cowger, Brian Ward, Gina Brown-Guedira, James K. M. Brown

**Affiliations:** ^1^U.S. Department of Agriculture – Agricultural Research Service, North Carolina State University, Raleigh, NC, United States; ^2^Department of Crop Genetics, John Innes Centre, Norwich, United Kingdom

**Keywords:** *Parastagonospora nodorum*, Septoria nodorum blotch, quantitative resistance, genotyping by sequencing, genome-wide association study, necrotrophic effectors, necrotrophic effector sensitivity genes

## Abstract

Important advances have been made in understanding the relationship of necrotrophic effectors (*NE*) and host sensitivity (*Snn*) genes in the *Parastagonospora nodorum*-wheat pathosystem. Yet much remains to be learned about the role of these interactions in determining wheat resistance levels in the field, and there is mixed evidence on whether breeding programs have selected against *Snn* genes due to their role in conferring susceptibility. SNB occurs ubiquitously in the U.S. Atlantic seaboard, and the environment is especially well suited to field studies of resistance to natural *P. nodorum* populations, as there are no other important wheat leaf blights. Insights into the nature of SNB resistance have been gleaned from multi-year data on phenotypes and markers in cultivars representative of the region’s germplasm. In this perspective article, we review the evidence that in this eastern region of the U.S., wheat cultivars have durable quantitative SNB resistance and *Snn–*NE interactions are of limited importance. This conclusion is discussed in light of the relevant available information from other parts of the world.

## Introduction

**Figure 1:**
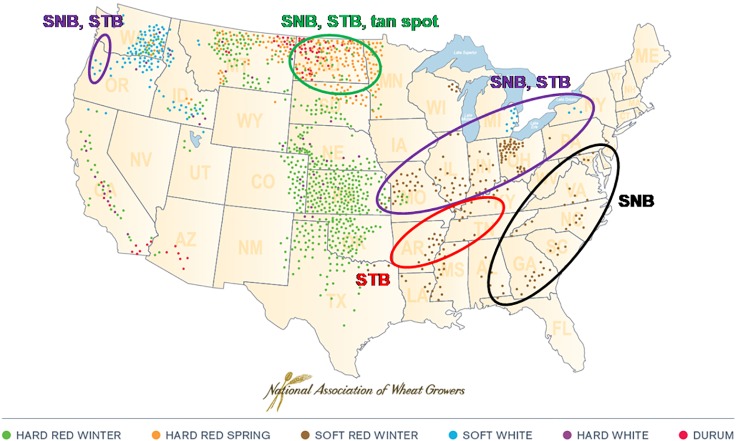
Septoria nodorum blotch (SNB), caused by *Parastagonospora nodorum*, co-occurs with Septoria tritici blotch (STB) (caused by *Mycosphaerella graminicola*) in the western, moist areas of the Pacific Northwest; the upper Plains states of North Dakota, South Dakota, and Minnesota; and the states adjacent to the Great Lakes. It is the only important wheat leaf blotch in the eastern seaboard states from Florida to Delaware. (Tan spot caused by *Pyrenophora tritici-repentis*).

Septoria nodorum blotch (SNB) of wheat, caused by the ascomycete fungus *Parastagonospora nodorum*, has a global distribution, although severe epidemics appear to be confined mainly to Australia, parts of northern Europe and the U.S., and parts of north Asia. In some areas such as the United Kingdom, SNB has become less significant than formerly as a result of successful breeding for resistance combined with reductions in atmospheric sulphur dioxide [discussed by (Arraiano et al., 2009); see also (Brown, 2015) and (Chandramohan and Shaw, 2013)]. In the U.S., SNB is a recurrent disease of wheat in several geographic regions (**Figure 1**). It co-occurs with Septoria tritici blotch in the western, moist areas of the Pacific Northwest; the upper Plains states of North Dakota, South Dakota, and Minnesota; and the states adjacent to the Great Lakes (Shaner and Buechley, 1995; Cowger et al., 2002; **Figure 1**). The only U.S. region where *P. nodorum* is the sole important leaf blotch pathogen of wheat is the eastern seaboard, making that environment ideal for field studies of SNB resistance and epidemiology (Cowger and Murphy, 2007).

SNB can cause significant yield losses in the U.S. and other countries (Shah and Bergstrom, 1993; Bhathal et al., 2003). In the southeastern U.S., SNB has been a frequent and sometimes damaging disease of winter wheat for many years (Nelson et al., 1974; Allingham and Jackson, 1981; Cunfer, 1998). Soft red winter wheat (SRWW) is the main market class of wheat bred and grown in the eastern U.S. (**Figure 1**), and the third largest wheat market class in the nation by production volume (after hard red spring and hard red winter wheats) (USDA Economic Research Service, 2019). Annual production of U.S. SRWW ranged between 6.5 million to 15.5 million tons (2.4 million and 5.7 million bushels) during the decade 2010 to 2019 (NASS, 2019). Given its low to medium levels of protein and soft endosperm, the primary uses of SRWW are in flatbreads, crackers, cakes, pastries and animal feed. Resistance to SNB is an ongoing breeding objective for SRWW (Cowger and Murphy, 2007).

## *Snn*–NE Interactions and Resistance

Wheat has nine named sensitivity (*Snn*) genes that interact with matching necrotrophic effectors (NE) of *P. nodorum* (Peters-Haugrud et al., 2019). The *Snn* genes have been found on wheat chromosomes 1AS, 1BS, 2DS, 2DL, 4BL, 5BS, 5DS, 5BL, and 6AL. These genes, which were detected as QTL using bi-parental mapping populations, interact with pathogen NE to increase the severity of SNB in what is known as an “inverse gene-for-gene” interaction. In wheat*-P. nodorum* and some other necrotrophic pathosystems, some sensitivity genes are apparently R-genes of the type that normally elicits resistance upon pathogen recognition, but have been hijacked by necrotrophic pathogens to elicit susceptibility (Nagy and Bennetzen, 2008; Faris et al., 2010; Gilbert and Wolpert, 2013; Shi et al., 2016). The NE are small secreted proteins, generally in the 10–30 kD range, that elicit programmed cell death when recognized by the host (Friesen and Faris, 2010). In the wheat-*P. nodorum* pathosystem, the interactions of *Snn* genes and NE may be incremental, unlike a classic gene-for-gene interaction; i.e., possessing two sensitivity genes can cause more susceptibility than possessing one, if the matching pathogen effectors are present (Chu et al., 2010; Abeysekara et al., 2012).

Of the wheat NE sensitivity genes investigated to date, *Tsn1* appears to be the one with the largest positive effect on susceptibility (Liu et al., 2006). *Tsn1* has features typical of pathogen recognition genes, including serine/threonine protein kinase (S/TPK) and nucleotide binding site (NBS) and leucine-rich repeat (LRR) domains (Faris et al., 2010). SnToxA is the NE that is recognized by Tsn1, while SnTox1 is recognized by Snn1 and SnTox3 by Snn3.

In an international comparison of *NE* frequencies and haplotypes, *SnToxA* was usually absent in the majority of *P. nodorum* isolates, depending on the country of origin, including in 75% of *P. nodorum* isolates from both spring and winter regions of the U.S. (Stukenbrock and McDonald, 2007; McDonald et al., 2013). An exception was Australia, but the evidence there appears to be mixed. Of 73 Australian *P. nodorum* isolates, 97% possessed *SnToxA* (Stukenbrock and McDonald, 2007; McDonald et al., 2013); however, that collection was from a single field and the *Tsn1* status of the wheat cultivar was unknown (Oliver et al., 2009). As over half of sampled wheat cultivars grown in Western Australia were sensitive to *SnToxA*, it appeared that breeders there had not selected against *Tsn1*, possibly because neither sensitivity to *SnToxA* nor to *SnTox3* was significantly associated with cultivar resistance level as measured *via* artificial inoculation with a mixture of current isolates (Oliver et al., 2009; Waters et al., 2011) By contrast, effector sensitivity tests on Vavilov wheat collection accessions showed that since 1940, breeders in Russia and Kazakhstan had selected directly or indirectly against *Tsn1* and also against *Snn1*, although not against *Snn3* (Phan et al., 2018).

The nine known *P. nodorum NE* and matching wheat sensitivity genes have been identified and characterized mainly using hard red spring or durum wheat lines and *P. nodorum* isolates from the upper Midwest state of North Dakota (references in Peters-Haugrud et al., 2019). It was recently discovered that the upper Midwest and eastern U.S. *P. nodorum* populations differ dramatically in the frequency of *SnToxA* (Richards et al., 2019). Specifically, Population 1 as examined by Richards et al. (2019) consisted of 105 P*. nodorum* isolates from hard spring and winter wheat and durum wheat in North Dakota, South Dakota, and Minnesota, while Population 2 included 67 isolates from the soft winter wheat states of the eastern U.S., stretching from Texas and Arkansas northeast to New York and south to Georgia. The frequencies of *SnToxA* were 96 and 4% in Population 1 and Population 2, respectively. That is, *SnToxA* was almost universal in the upper Plains isolates, and almost entirely absent in the eastern soft wheat isolates. The populations did not differ greatly for the other analyzed NEs: *SnTox1* occurred at frequencies of 100 and 88% in Populations 1 and 2, respectively, and *SnTox3* at frequencies of 62 and 52%, respectively. These results are consistent with a smaller previous study showing that *SnToxA* and *SnTox3* were present only in a minority of isolates from the eastern U.S. *P. nodorum* population (15 and 39%, respectively), but *SnTox1* was present in 74% (Crook et al., 2012).

On the host side, a sample of 26 wheat genotypes from eastern U.S. wheat breeding programs was surveyed to determine the presence of the *Tsn1*, *Snn1*, and *Snn3* sensitivity genes (Bertucci et al., 2014). *Tsn1* was not found in any of 12 eastern U.S. soft winter lines. However, the gene was present in 6 of 11 hard winter wheat cultivars, particularly those with origins in the southern Plains states of Nebraska, Kansas, Oklahoma and Texas, that had been utilized for hardness and other traits in a new program to breed bread wheats for the eastern U.S. market. Further, the sensitivity gene *Snn3* was present in 7 of the 12 soft wheat cultivars, and 6 of the 11 hard wheat cultivars, while *Snn1* was absent from all lines. In North America, *Snn1* is thought to be less frequent in hexaploid than in durum wheat germplasm (J. Faris, personal communication.) The evidence from these studies suggests that variation in susceptibility of SRWW could be affected by *Snn3*, but not by *Tsn1* and probably not by *Snn1*. From the small sample of hard winter wheats with origins in the central-southern Great Plains (Nebraska, Kansas, Oklahoma, and Texas), it appears that *Tsn1* may play a role in susceptibility there.

The hypothesis that *Tsn1* is rare in eastern U.S. soft wheat germplasm was reinforced by examining data from available eastern wheat germplasm. A dominant Kompetitive Allele-Specific Polymerase chain reaction (KASP) marker (primer sequences Tsn1_AL1 5ʹ-GAAGGTCGGAGTCAACGGATTCTATTCGTAATCGTGCCTTCCGG-3ʹ and Tsn1_C1 5ʹ-CTGCCCTTCACTTAGCCTGTCAC-3ʹ) was developed based on the published *Tsn1* gene sequence (Faris et al., 2010). Frequency of *Tsn1* was assessed using DNA of a panel of 330 eastern wheat lines that included ten 19^th^ century landraces, 188 cultivars developed from 1915 to 2011, and 132 elite experimental lines. *Tsn1* was detected in only 15 samples, including seven soft wheat cultivars and two experimental lines. Six of the 15 eastern hard winter wheat breeding lines in the panel possessed the gene. None of the soft winter wheat landraces had *Tsn1* and there was only one *Tsn1*-positive line developed prior to 1950, suggesting that *Tsn1* was either rare or absent in foundational germplasm of the eastern soft wheat growing region.

Although this panel was not an exhaustive sampling of historical germplasm, this conclusion is supported by the low frequency of *Tsn1* in later cultivar releases. Starting in 2013, the *Tsn1* marker was routinely analyzed on advanced wheat experimental lines and released varieties serving as checks in the elite Uniform Eastern and Uniform Southern SRWW nurseries operated by the U.S. Department of Agriculture-Agricultural Research Service. Entries in these nurseries have a high likelihood of subsequent release as commercial varieties. Through 2019, *Tsn1* marker data were available for 350 lines, which came from public and private breeding programs in states throughout the eastern U.S. soft wheat area, from Texas to Michigan and eastward. Only 9% carried the *Tsn1* marker. With the exception of cv. Bess (PI 642794), the 32 *Tsn1*-bearing genotypes were all experimental lines, and it is unknown how many were subsequently released for commercial use.

Taken together, the information suggests that *Tsn1* probably occurs at relatively high frequency in wheat cultivars planted in North Dakota, South Dakota, and Minnesota, given the high frequency of *SnToxA* in the corresponding *P. nodorum* population, and thus *Tsn1* may contribute to variation in SNB susceptibility in wheat crops in that region. *Tsn1* may also be common in hard winter wheats from central states farther south, such as Oklahoma and Kansas. However, *Tsn1* is rare in eastern U.S. commercial SRWW cultivars. It appears to have been nearly absent from the foundational germplasm of that market class, and although the gene was subsequently introduced into SRWW, it has remained rare, perhaps due to phenotypic selection for SNB resistance. Other sensitivity genes, in particular *Snn3*, could be more widely present in eastern U.S. wheat production.

The efficacy of such phenotypic selection is best illustrated in the U.K. There, breeding for SNB resistance succeeded in reducing the disease to inconsequential levels (Scott and Benedikz, 1977; Scott et al., 1982; Brown, 2015) despite the absence of relevant information about *Snn* genes until a recent survey. In a panel of 480 northwest European varieties, of which approximately 330 were from the U.K., sensitivity to SnTox3 was more common than that to SnTox1, while sensitivity to SnToxA was rare (Downie et al., 2018). The relative contributions of *Snn* genes and other, minor genes to susceptibility remain to be investigated in the U.K.

In contrast with the findings of Downie et al. (2018), SnToxA sensitivity was more common in Scandinavian spring wheats: among 157 genotypes, the majority Norwegian and Swedish and the rest from CIMMYT, sensitivity to SnToxA was present in 45% (Ruud et al., 2018). In keeping with this relatively high frequency of SnToxA sensitivity in Scandinavian spring wheats, 69% of a sample of Norwegian *P. nodorum* isolates had the *SnToxA* gene; the frequencies of *SnTox1* and *SnTox3* were 53 and 76%, respectively (Ruud et al., 2018). The higher *SnToxA* frequency is the main difference with the only other survey to date of *NE* in western European *P. nodorum*: there, frequencies were reported of about 12% *SnToxA*, 85% *SnTox1*, and 65% *SnTox3* in samples mainly from Switzerland, Sweden, and Denmark (McDonald et al., 2013).

Ruud et al. (2018) found some association between field susceptibility and sensitivity to SnToxA, but not to either of the other NE. This is to date the only evidence that *Snn* and *NE* frequencies are factors explaining SNB resistance in European wheat production. In other words, it appears that SnToxA-Tsn1 interactions are important in Norway, likely because the prevalence of *Tsn1* in spring wheat germplasm has selected for a relatively high frequency of *SnToxA* in the pathogen population. There is no evidence that SnToxA-Tsn1 interactions or other *Snn*–NE interactions are important in other, primarily winter-wheat producing regions of western Europe.

Available information from Australia also leads to the conclusion that NE sensitivity is not closely related to disease phenotype in the field. Neither individual sensitivity to *SnToxA*, to *SnTox1*, or to *SnTox3* was consistently significantly associated with field disease resistance scores of representative Australian wheat cultivars in more than one year (Tan et al., 2014). A *P. nodorum* strain lacking *SnToxA*, *SnTox1*, and *SnTox3* was found to produce one or more novel NE in culture filtrates, but there was no clear relationship between sensitivity to that new NE and SNB susceptibility in 46 tested cultivars with varying degrees of resistance (Tan et al., 2015). Only by pooling scores of sensitivity to *SnToxA*, *SnTox1*, and *SnTox3* and the novel NE did the authors detect a significant association between NE sensitivity and field disease score in the 46 cultivars, and even then a quarter of the more susceptible lines had low pooled NE sensitivity and about 40% of more resistant lines had high pooled NE sensitivity (Tan et al., 2015).

Given the above, what is known about the genetics of wheat resistance to SNB? The Tsn1-SnToxA interaction can confer susceptibility in the field in regions where *Tsn1* has not been selected against. However, contrary to what is sometimes stated [e.g., (Waters et al., 2011; Tan et al., 2015; Phan et al., 2018)], there is no reason to suppose that in general, wheat-*P. nodorum* interactions are completely or even mainly defined by *Snn–*NE relationships, or that NE are required for pathogenesis. Resistance is polygenic, involving genes with minor effects, and foliar and glume infections are controlled by genes that segregate independently (Fried and Meister, 1987; Bostwick et al., 1993; Francki, 2013; Francki et al., 2017). While some of these minor interactions may involve NE and *Snn* genes, whether or not they have been characterized, others may well not.

## Capitalizing on Long-Term Screening Data

Resistance to SNB is quantitative, and we sought evidence on its durability in commercial wheat production in the eastern U.S. If *Snn–*NE interactions were common in this region, one might expect cultivars would decline in quantitative resistance (QR) as common *Snn* genes exerted a selective effect by increasing the frequency of matching NE. To evaluate this hypothesis, we took advantage of data from a long-term resistance screening nursery to assess durability and to conduct a genome-wide association study (GWAS); both analyses are described below.

Between 2009 and 2018, 2,161 wheat genotypes (the vast majority SRWW) were phenotyped for SNB severity in the Eastern SNB Nursery of the USDA, the goal of which is to increase SNB resistance in the germplasm of participating breeding programs. Replicated field trials were performed in 16 year* location environments in North Carolina; in most years there were two locations, but in a few years there was only one due to crop or disease failure. Most genotypes were advanced experimental lines from 21 breeding programs in the central and eastern U.S., and were in the nursery fewer than 3 years; however, some commercial varieties were tested longer.

Genotypes were grown in plots consisting of two adjacent 1.3-meter rows, with two replicate plots per location. Each year, wheat straw infected by the natural *P. nodorum* population of North Carolina was spread evenly on the plots at Zadoks growth stage 25-29 (Zadoks et al., 1974; prior to stem elongation) to provide inoculum (Cowger and Murphy, 2007). The pathogen population in the nursery was therefore expected to broadly reflect the composition of that in North Carolina generally. During heading and anthesis, large-droplet irrigation was applied at some sites to enhance disease development and ensure inoculum dispersal to all upper plant parts, minimizing infection escape due to tall stature. Disease severity was rated using a 1–9 scale on a whole-plot basis at Zadoks growth stage 75–77 (medium to late milk stage of grain filling) for foliar and glume symptoms separately. Earlier-maturing lines were scored earlier than late-maturing lines, and no significant relationship was detected between SNB severity and heading date. Significant differences among genotypes were observed (https://www.ars.usda.gov/southeast-area/raleigh-nc/plant-science-research/docs/nursery-reports/page-6/).

### Statistical Evidence of Durability of Eastern US Wheat QR to SNB

**Figure 2:**
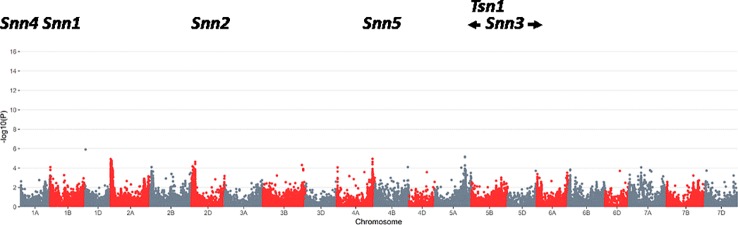
Results of a genome wide association study using SNPs and foliar severity of Septoria nodorum blotch on 1,231 wheat lines from the eastern U.S. during the period 2009 to 2018. No significant quantitative trait loci were detected for foliar resistance (false discovery rate threshold of 0.05), whereas a significant QTL was separately detected for glume resistance (data not shown; report in preparation).

Large longitudinal datasets on varieties in official trials over years or decades are an underutilized resource that can provide insight into advances in breeding crop varieties for diverse traits (Brown et al., 2019; **Figure 1** in Cowger and Brown, 2019). In the present case, to assess durability of QR to SNB, a linear mixed model was fitted to whole-plot SNB severity data from 19 SRWW cultivars that were tested at least 7 years each in the Eastern SNB Nursery trials, and one genotype (P26R61) that appeared 5 years in the trials and was known to possess QR to multiple diseases (Cowger and Brown, 2019, **Figure 2**). Variety and the linear effect of year were fixed effects and the interaction of variety and year was a random effect. While mean levels of foliar and glume SNB, estimated across all cultivars, increased significantly in the nursery over the period due to improved screening methodology such as irrigation (*P* < 0.002), there was only weak evidence for variation in the rate at which foliar SNB severity changed on cultivars with respect to each other (*P* = 0.06), and no such evidence with regard to glume SNB, demonstrating the durability of QR in this germplasm [for further details, see (Cowger and Brown, 2019)]. This degree of durability would not be expected if a large proportion of resistance were controlled by genes involved in specific interactions with *P. nodorum* genotypes, such as those for NE sensitivity.

### Genome-Wide Association Study Reveals No Significant Loci for Foliar QR

A mixed-model GWAS was conducted on 1,231 of the lines phenotyped in the Eastern SNB Nursery from 2009 to 2018 for which genotyping-by-sequencing (GBS) data were available. The 1,231 lines were mainly from two regional nurseries: the Gulf Atlantic Wheat Nursery (GAWN), which comprises wheat lines from southern soft wheat states, and the Mason-Dixon Nursery, which includes wheat lines from the more northerly soft wheat states of Kentucky, Virginia, North Carolina and Maryland.

The GBS data included 54,856 SNPs. Briefly, genomic DNA was isolated from seedlings at the two-leaf stage. GBS library preparation was performed on genomic DNA according to Poland et al. (2012). The Burrows-Wheeler Aligner (Li and Durbin, 2009) v0.7.17 was used to align single-end Illumina reads to the Chinese Spring IWGSC RefSeq v1.0 wheat reference genome (International Wheat Genome Sequencing Consortium, 2018). Variant identification, SNP filtering and imputation were as described in Ward et al. (2019). The first five genotypic principal components, calculated for the SNP matrix using EIGENSTRAT (Price et al., 2006), were used to model and account for population structure, i.e., relatedness of genotypes. The Genome Wide Complex Trait Analysis (GCTA) software (Yang et al., 2011) was used to perform single-locus mixed linear model GWAS and to estimate the proportion of phenotypic variance explained by all SNPs jointly.

The GWAS revealed no significant QTL for foliar resistance to *P. nodorum*, including no significant effects in chromosomal regions near the *Tsn1*, *Snn1*, or *Snn3* loci [at a false discovery rate (Benjamini and Hochberg, 1995) threshold of 0.05; **Figure 2**]. This was consistent with the low frequencies of *Tsn1* and *SnToxA* found in SRWW and the southeastern *P. nodorum* population, respectively. It was notable that *Snn3* did not contribute significantly to foliar resistance in the GWAS, despite the intermediate frequencies of *Snn3* and *SnTox3* in the host and pathogen populations. This suggests that SNB resistance in the study area is primarily determined by genes other than those conferring NE sensitivity, although it is possible that as-yet undetected *Snn*–NE pairs are playing very small roles.

It should be noted that a GWAS using the natural *P. nodorum* population is different from one designed to detect genes when only a fraction of either host or pathogen diversity is present. In other words, the use of one or a few isolates would give greater power to detect resistance loci that are effective against a specific set of pathogen genotypes, depending also on the host genes being tested. However, such a GWAS would also have to be done under controlled conditions to avoid confounding inoculum. The results would not be reflective of field performance of tested genotypes, whereas the present question was precisely whether Snn-SnTox interactions significantly influence field performance of regionally representative germplasm in the presence of the full range of pathogen diversity. In fact, the same GWAS that did not detect foliar QTL did detect a new major QTL for glume resistance, and a separate report on the novelty of that locus is in preparation. The foliar QR to SNB in eastern U.S. wheat germplasm appears to be governed by numerous loci, each with a small effect, consistent with previous studies in this region (Nelson and Gates, 1982; Bostwick et al., 1993), and with durable resistance to other foliar diseases of cereals (Brown, 2015).

## Conclusion

The above evidence leads to two conclusions:

(1) Regional/national differences in *SnToxA* frequency are likely to mirror the frequency of *Tsn1* in the wheat germplasm that is widely deployed in each area. Globally, evidence on whether breeders have selected against *Tsn1* or other *Snn* genes is mixed and regionally specific. In commercial wheat production environments that are conducive to SNB, extensive use of *Tsn1* in widely planted cultivars appears to have selected a high frequency of *SnToxA* in the *P. nodorum* population. Also, it may be that due to germplasm histories, spring wheat areas are more apt to have high *Tsn1* and *SnToxA* frequencies than winter wheat areas, provided conditions are conducive to SNB. At the same time, *SnToxA* appears to exact a fitness cost, as it has a low frequency in the pathogen population where *Tsn1* is selected against in current wheat varieties.

In the U.S., it appears there are important regional differences with regard to *Tsn1*. Eastern SRWW breeding programs started with a germplasm base in which *Tsn1* was all but absent, and had apparently selected against the gene even before marker data became available in 2013, in part because SNB symptoms have been more ubiquitous, frequent and unconfounded by other leaf blotches than in other U.S. regions. Hard wheat breeding programs in the central U.S. states have apparently retained *Tsn1*, perhaps because it is a deliberately selected resistance gene and/or is linked to important agronomic traits such as hardness, and because phenotypic selection is hampered by infrequent occurrence of SNB epidemics.

(2) *Snn–*NE interactions do not appear to be an important factor in the QR observed in eastern U.S. soft wheat germplasm. The QR in this germplasm is evidently stable and relatively durable, and there is no evidence that the pathogen population is being strongly selected for aggressiveness as one might expect if *Snn* genes were exerting a substantial influence. Surprisingly, while *Snn3* has been detected in soft wheat germplasm, the GWAS yielded no evidence that *Snn3* by itself was important in explaining resistance variation, similar to findings in Norway and Australia. It is possible that as-yet undetected *NE* and *Snn* genes are playing a role in the host-pathogen relationship in the eastern U.S. It is also possible that as in other crop diseases (Cowger and Brown, 2019), this QR is governed by many genes with small effects that are not directly involved in *Snn–*NE interactions. A field screening program that utilizes the natural *P. nodorum* population of an area is an essential tool for selecting against susceptibility, whether *Snn*-linked or not.

## Data Availability Statement

The datasets generated for this study are available on request to the corresponding author.

## Author Contributions

CC contributed field data, BW conducted GWAS, GB-G contributed marker data, and JB performed statistical analysis. CC led manuscript production and all authors contributed text and edited manuscript.

## Funding

The research of CC, BW, and GB-G is funded by the United States Department of Agriculture-Agricultural Research Service (USDA-ARS), an equal opportunity provider and employer. The research of JB is supported by the Biotechnology & Biological Sciences Research Council Plant Health strategic programme.

## Conflict of Interest

The authors declare that the research was conducted in the absence of any commercial or financial relationships that could be construed as a potential conflict of interest.

## Abbreviations

NE, necrotrophic effectors; GWAS, genome-wide association study; GBS, genotyping by sequencing; *Snn* gene, host sensitivity gene; QR, quantitative resistance; QTL, quantitative trait locus; SNP, single-nucleotide polymorphism; SNB, Septoria nodorum blotch; SRWW, soft red winter wheat; USDA, United States Department of Agriculture.
